# Knowledge and perception of HPV vaccination among Lebanese mothers of children between nine and 17 years old

**DOI:** 10.1186/s12978-024-01764-7

**Published:** 2024-03-27

**Authors:** Nassif Elissa, Hadchity Charbel, Azzi Marly, Nader Ingrid, Saleh Nadine, Abdo Rachel

**Affiliations:** 1 Faculty of Medical Sciences, Lebanese University, Hadath, Beirut, Lebanon; 2 Faculty of Public Health, Lebanese University, Beirut, Lebanon; 3 INSPECT-LB Institut National de Santé Publique, Epidémiologie Clinique et Toxicologie-Beirut, Beirut, Lebanon

**Keywords:** Awareness, Cervical cancer, Human papillomavirus (HPV), Knowledge, Lebanon, Mothers, Parents, Vaccine

## Abstract

**Background:**

The human papillomavirus (HPV), a prevalent sexually transmitted infection, is linked to a wide range of diseases, with cervical cancer being the most common and serious one. HPV vaccination is crucial for preventing cervical cancer and other HPV-related problems. The low acceptability of HPV vaccination among teenagers globally is largely due to a lack of understanding and information about HPV among parents. Our study aimed to evaluate the level of knowledge, attitude, intention, and HPV vaccination among parents in Lebanon as well as the variables influencing Lebanese mothers' intentions to vaccinate their children.

**Methods:**

A cross-sectional survey-based study involving 392 participants was conducted between May and June 2022. The study assessed parents' intention to vaccinate their children against HPV, their knowledge about HPV, and the HPV vaccine. The data was collected through an anonymous electronic questionnaire. A bivariate analysis was conducted using Student t-test and ANOVA to examine the relationship between the dependent variable “Intention to vaccination” and the secondary variables. The level of statistical significance was set at 0.05 for all data.

**Results:**

Our findings showed that only 63% of the 392 participants claimed they would give their child the HPV vaccination. A positive significant association was demonstrated between "Intention to vaccinate against HPV" and mother's nationality, father's educational level, family income per month, information received about the HPV vaccine, parents' HPV vaccination, insurance coverage of the HPV vaccine, children's vaccinations with all required vaccines, knowledge of HPV, and knowledge of the HPV vaccine. Furthermore, when parents know about HPV, their desire to vaccinate their child increases by a factor of 1.832 times, and by 1.207 times when their knowledge level increases by one point.

**Conclusion:**

The majority of parents lacked a general understanding of most HPV-related statements, which highlights the requirement for educational interventions to raise parental awareness, understanding, and attitudes toward HPV and, as a result, increase parental acceptance of vaccinating their children. To increase the vaccination rate among adolescents, government authorities should ensure that the HPV vaccine is available in all hospitals and clinics and should be provided free of charge.

## Introduction

Human papillomavirus (HPV) is one of the most common sexually transmitted viruses in the world; it represents a severe public health concern [1].

HPV is a non-enveloped, double-stranded, circular DNA virus belonging to the Papillomavirus family. The most typical sites of infection for many viruses and bacteria, including papillomaviruses, are mucosa and skin. Moreover, HPV reaches the epithelium through skin/mucosa lesions and infects basal epidermal stem cells [1].

More than 200 subtypes of HPV have been identified based on DNA sequence data revealing genetic variations, and 85 HPV genotypes have been thoroughly studied [2].

HPV is the driving factor behind several epithelial infections and cancers, mostly on the cutaneous and mucosal sites. It can be divided into high-risk (types 16, 18, 31, 33, 34, 35, 39, 45, 51, 52, 56, 58, 59, 66, 68, 70) and low-risk (types 6, 11, 42, 43, and 44) varieties relying on their association to precursor lesions and cervical cancer [2].

In fact, HPV infection causes major health concerns, such as anogenital malignancies (cervical, vaginal, vulvar, anal) and oropharyngeal cancers caused by high-risk HPVs, and anogenital warts brought on by low-risk HPVs [3]. Although HPVs are known to contribute to the growth of different malignancies, HPV infections are frequently asymptomatic, recover spontaneously, and are temporary [4].

A global sexually transmitted infection surveillance report published in 2015 examined the prevalence of the most common sexually transmitted infections, including HPV. In this report, WHO reported that over 290 million sexually active women will get infected with HPV [4].

Moreover, the Center for Disease Control and Prevention determined in their latest research in 2018 that the prevalence of genital HPV in people aged 18–59 was approximately 45.2% in males and 39.9% in women [5]. According to prior studies, the prevalence of HPV among women with normal cervical cytology (NCC) was 10.4% and 11.7%, respectively, in 2007 and 2010, and has decreased to 9.9% in 2019 [1].

The large intra-continental variation in HPV prevalence permits global regional repartition. The HPV distribution profile in women with NCC is almost identical to that of the general female population in terms of the geographical global areas [6]. The prevalence of HPV is greater in developing nations. For instance, HPV prevalence was greater in Sub-Saharan Africa (SSA) (24.0%), particularly in the areas of Eastern Africa (33.6%) and Latin America, according to a compilation of studies from cytologically healthy women [6, 7]. The highest HPV incidence in all females was reported in Asian regions. HPV prevalence was low in practically all European nations (< 30%), as well as in Western Europe (3.7%). As a result, HPV infection rates in developing nations (42.2%) are greater than in developed regions (22.6%).

Cervical cancer has the second-highest mortality rate among women globally after breast cancer [1] and has been identified as a life-threatening condition. Indeed, it has many complications resulting from the disease itself or from the treatment. The complications can range from the very trivial, such as minor vaginal bleeding, excessive urination, pain, menopause, and anxiety, to the potentially lethal, such as severe hemorrhage, renal failure, and kidney failure [8].

In 2020, cervical cancer ranked seventh among all cancers and first among gynecologic cancers [9]. Moreover, Global Cancer Incidence, Mortality and Prevalence (GLOBOCAN) estimates that there were 311,000 fatal cases and 570,000 new cases of cervical cancer globally in 2018. If the right measures are not taken into account, the global prevalence of cervical cancer is expected to increase by 21% and 27% of cases and deaths, respectively [10].

According to projections for 2020 In Lebanon, around 124 new cases of cervical cancer are detected each year. Moreover, cervical cancer was demonstrated to be the tenth most common cause of female cancer in Lebanon and is the eighth most prevalent female malignancy among women aged 15 to 44 years [11].

Compared to non-HIV-infected women, HIV-positive women are 6 times more likely to develop cervical cancer [12]. In addition, the highest incidence of HPV is observed among teens and young adults between the ages of 15 and 25, and it is estimated that more than 75% of new HPV infections occur in this age group [13]. Consequently, it is highly important to introduce HPV vaccination as a strategy for preventing cervical cancer and other problems associated with HPV [2].

Three preventive HPV vaccines have been licensed for use worldwide: bivalent, quadrivalent, and monovalent. The quadrivalent vaccination protects against four HPV types (6, 11, 16, and 18), whereas the bivalent vaccine protects against two HPV types (16 and 18). In addition to the types covered by the quadrivalent vaccination, a recently released monovalent vaccine protects against five HPV types 31, 33, 45, 52, and 58 [14].

The Center of Disease Control and Prevention (CDC) and its Advisory Committee on Immunization Practices (ACIP) have indicated that regular HPV vaccine is recommended for boys and girls at the age of 11 or 12 years, it can start at the age of nine and continue until the age of 26 years [15].

Despite an increase in HPV vaccines, many teenagers have not got the entire series. According to the Centers for Disease Control and Prevention, around 66% of teenagers aged 14–17 years received the first dose in the immunization series in 2017, but fewer than half received all of the necessary doses to complete the series. This suggests that 51% of adolescents who have gotten one HPV series dosage have not received the subsequent doses [16].

HPV vaccination has been shown to be limited worldwide [7]. Notably, one of the main factors responsible for low HPV vaccination uptake is a general lack of awareness and knowledge about HPV infection and its outcomes [17].

Various general communication approaches could be accessible to everyone to provide knowledge about HPV, HPV vaccine, and HPV-related diseases, such as written awareness (posters, booklets…), media campaigns (radio, television programs, online educational pages…), and verbal communication (demonstrations from a healthcare professional) [17]. Additionally, HPV awareness programs have been launched in schools as an approach to raising awareness among adolescents. Even though these programs primarily target teenagers (ages nine to 13), they have been extremely effective in achieving notable improvements in HPV awareness as well as sexual behavior and relationships [18].

Given the young age at which vaccinations are administered, parents are typically the main decision-makers, and parental approval is required for vaccination uptake. To that purpose, educational HPV initiatives have focused mainly on educating parents rather than teenagers to raise vaccination rates [17, 19, 20].

When it comes to their children's health, moms are usually the main decision-makers [19]. To this end, many studies have been conducted to assess maternal HPV knowledge and acceptance of HPV vaccination. In addition, it seems important to identify factors that influence a mother's knowledge of HPV, which allows these factors to be addressed in the future in order to increase the vaccination rate.

AZH et al. conducted a cross-sectional research on 126 mothers having daughters aged 12 to 14 years to assess the factors influencing mothers’ knowledge and intention to vaccinate their children. Even though half of the moms had a diploma, it was reported that the mothers' knowledge and intention about HPV were insufficient. Moreover, half of the mothers who were familiar with the vaccine were concerned about its negative effects, and the majority of moms thought immunization should be postponed until after their daughters get married [21]. Their research revealed that knowledge and some socioeconomic factors affected mothers' intentions, highlighting the responsibility of medical professionals in educating mothers and raising their awareness about HPV and HPV vaccination.

According to research vaccination programs are well-followed until completion in the majority of developed countries. However, vaccination program implementation is limited in underdeveloped countries, which contributes to the reported high morbidity due to HPV infection and HPV infection-related disorders. The limitations of HPV vaccinations in these regions include poor living conditions, co-infections with other diseases, inadequate medical services, parents who refuse to vaccinate their children, delayed implementation of the vaccine program, and high vaccine costs [1].

According to local research, 2.5% and 16.5% of female Lebanese schoolgirls in a small sample of 215 students at a top private school in Beirut had received the HPV vaccine [19, 22]. Unfortunately, there is a serious paucity of research on the issue, and the accessible studies primarily focus on particular populations. The HPV vaccination rate is limited in Lebanon among eligible girls. Hence, it is necessary to evaluate the factors leading to this low rate and find the gaps that must be addressed through awareness programs to increase the vaccination rate.

In Lebanon, Abou el Ola et al. showed that there are several obstacles to HPV vaccination, the most significant of which is the mothers' lack of information about HPV, cervical cancer, and prevention strategies [19].

Few researchers have explored adolescent mothers' attitudes and intentions to vaccinate their children especially their daughters [21, 23]. Despite the seriousness of HPV-related diseases mainly cervical cancer and the importance of HPV vaccination among young girls, few studies have been conducted in the Middle East to evaluate the knowledge of mothers about HPV and their acceptability of HPV vaccination [19].

Unfortunately, Lebanon lacks formal national recommendations for cervical cancer screening routines and a national HPV vaccination campaign [24].

After reviewing the literature, we discovered a remarkable scarcity of research papers addressing HPV and its vaccination in the Lebanese community. In order to fill in any gaps in the literature, we conducted this study to evaluate the level of knowledge, attitude, intention, and HPV vaccination among teenagers in Lebanon, as well as the factors influencing Lebanese mothers' intentions to vaccinate their children.

## Methodology

A cross-sectional study was conducted in 2022 between the 1st of May and the 30th of June 2022. The study included parents of students aged between nine and 17 years to assess their intention to vaccinate against HPV and their knowledge about HPV and HPV vaccine.

In May 2022, we conducted a pilot test including children’s parents to include them in the study. A request form was sent to parents. The form was designed on Google Form (electronic survey) to get their approval to participate in the study and therefore include them. For the pilot test participants found that the survey questions were clear and easily understandable, indicating that the language used in the questionnaire was appropriate. The average time taken by participants to complete the survey was within an acceptable range, suggesting that the survey was not overly time-consuming. Additionally, all questions were deemed relevant by the majority of participants, indicating that the survey instrument effectively covered the necessary aspects of the research topic. As a result, no adjustments were made to the survey instrument based on the pilot test results, affirming its suitability for our research study. This positive feedback from the pilot test participants validates the robustness of our survey instrument and instills confidence in its ability to collect meaningful data for our study.

The survey was distributed to all participants through social media platforms using the snowball sampling technique. A request form was sent to parents, seeking their approval to participate in the study.

Different eligibility criteria were followed. This study included parents who are aged ≥ 18 years; are able to speak, read, and write English or French; being residents in Lebanon; being mother or a legal guardian of a children aged between 9 and 17 years who did or did not receive the HPV vaccine (if a parent had more than one children, they were asked questions about their eldest children).

Referring to the minimum sample size formula, n = Z2 × P(1 − P)/d2, the minimum required sample was 323 using an expected willingness of HPV vaccination of 30% based on the literature averages. A 95% confidence interval (Z = 1.96) and a 5% alpha error (d = 0.05) were selected for statistical significance.

The survey instrument was a self-administered anonymous electronic questionnaire. Each participant received a study code. The survey had been designed on an electronic form using Google forms and was available in both English and French, allowing participants to choose their preferred language.

Ethical considerations were taken into account throughout the study. Study was conducted to ensure participant confidentiality and informed consent. All data were anonymized to protect participant identities.

The survey includes four sections. The first section considered the demographic characteristics: mother’s age, mother’s nationality, father’s age, father’s nationality, residency governorate, educational level, profession, marital status, family income per month, and religion. Also the knowledge about Human Papilloma Virus (HPV) (Have you ever heard of the Human Papilloma Virus (HPV) vaccine?) and source of information.

The second section is designed to collect all information about the child: sex of the child, child’s age, number of siblings, child school grade, health coverage, completed vaccination, and vaccination against HPV. However, the third section of the survey consists of 23 items that cover knowledge about HPV and HPV vaccine. The items are distributed between 16 items concerning HPV-related knowledge (The response options for the 16 items were “true,” “false,” and “I don’t know”) [25]; and 7 items concerning HPV vaccine-related knowledge (The response options for the 7 items were “true,” “false,” and “I don’t know”). And the last section of the survey was designed to assess the intention to vaccinate: Mothers will be requested to answer if they will provide their child with the vaccination against HPV “Are you willing to vaccinate your child?” (The response options were Yes / No).

The Statistical Package for the Social Sciences (SPSS) version 26 was for data analysis. Basic descriptive statistics and frequency calculations were performed on all variables. Nominal variables were presented by frequency and proportions. Continuous variables were presented by mean, standard deviation, minimum and maximum values.

Intention to vaccination was presented by frequency and proportion (Willing or Not willing). Knowledge about HPV and HPV vaccination were presented as scores where the scores were computed by adding the sum of the items related to knowledge (correct answers will get a code “1” and incorrect answer will get a code “0”). The scores were presented by mean, standard deviation, minimum, and maximum value.

Bivariate relationship was enrolled between the dependent variable “Intention to vaccination” and the secondary variables (demographics, Knowledge about HPV, Knowledge about HPV vaccine, Information about HPV and all the secondary variables). Tests used in the bivariate settings were student t-test and ANOVA test. The level of statistical significance was set at 0.05 for all data.

A binary logistic analysis was enrolled to assess the factors predicting the intention to vaccination, and the model included all the factors statistically associated with the intention to vaccination in the bivariate settings.

## Results

Table 1 outlines the socio-demographic parameters of 392 participants included in the study. The mean age of mothers was 42.5 ± 5.4 years and the mean age of fathers was 47.8 ± 5.8 years. The majority of mothers (95.9%) and fathers (94.9%) were Lebanese. Concerning the educational level of mothers, 48.7% had a university diploma, 21.7% had higher education, and 29.6% had less than the university level. Concerning the educational level of fathers, 42.6% had a university diploma, 16.8% had higher education, and 40.6% had less than the university level. The majority of mothers (83.4%) and fathers (93.4%) were working in non-medical fields. Regarding their religion, 86% of mothers were Christians, 14% of mothers were Muslims, 85.5% of fathers were Christians, and 14.5% of fathers were Muslims. Participants were living in Beirut (25.5%), Mount Lebanon (60.7%), and other governorates (13.8%). The majority of participants were married (93.9%) and 6.1% were widowed. Regarding their family income, 46.2% received more than 8 million LBP, 26.5% received between 4 and 8 million LBP, and 27.3% received less than 4 million LBP.

**Table 1:** Demographic characteristics of the study population (N = 392)

		Frequency	Percent
Mother's age	Mean (SD)	42.5 (5.4)	
	Median	42	
	Min–Max	22–55	
Father's age	Mean (SD)	47.8 (5.8)	
	Median	48	
	Min–Max	25–60	
Mother's nationality	Lebanese	376	95.9
	Others	16	4.1
Father's nationality	Lebanese	372	94.9
	Others	20	5.1
Mother's educational level	No formal education	22	5.6
	School-level education	94	24
	University Diploma	191	48.7
	Higher Education (Masters, PhD)	85	21.7
Father's educational level	No formal Education	31	7.9
	School- level Education	128	32.7
	University Diploma	167	42.6
	Higher Education (Masters, PhD)	66	16.8
Mother's profession	Medical	65	16.6
	Non-medical	327	83.4
Father's profession	Medical	26	6.6
	Non-medical	366	93.4
Mother's religion	Christian	337	86
	Muslim	55	14
Father's religion	Christian	335	85.5
	Muslim	57	14.5
Residence	Beirut	100	25.5
	Mount Lebanon	238	60.7
	South Lebanon	13	3.3
	North Lebanon	5	1.3
	Beqaa	36	9.2
Marital status	Married	368	93.9
	Widow	24	6.1
Family income per month	Less than 2.000.000	29	7.4
	Between 2.000.000–4.000.000	78	19.9
	Between 4.000.000–8.000.000	104	26.5
	More than 8.000.000	181	46.2

Out of 392 participants, 20.7% had a history of cancer, 4.3% had a history of cervical cancer, and 7.9% had a family history of cervical cancer as shown in Table 2.

**Table 2:** History of cervical cancer in the study population (N = 392)

		Frequency	Percent
Do you have any history of cancer	No	311	79.3
	Yes	81	20.7
If your previous answer is yes, is it a cervical cancer	No	64	16.3
	Yes	17	4.3
	No history of cancer	311	79.3
Do you have any history of "cervical" cancer in your family	No	361	92.1
	Yes	31	7.9

Moreover, we evaluated the awareness about HPV and the rate of vaccination among participants. The results represented in Table 3 demonstrated that out of 392 participants, 231 (58.9%) had ever heard of the Human Papillomavirus (HPV) vaccine. The top four sources of information were Pediatrician (32.5%), physician (22.9%), Media/Internet (19.9%), and family (10%).

**Table 3:** Awareness about HPV in the study population (N = 392)

		Frequency	Percent
Have you ever heard of the Human papillomavirus (HPV) vaccine	No	161	41.1
	Yes	231	58.9
If your previous answer is yes, what is the source of these information?	Media/Internet	46	19.9
	Your physician	53	22.9
	Pediatrician	75	32.5
	Family	23	10.0
	Friends	12	5.2
	Others	19	8.2
	Did not hear of the HPV vaccine before	3	1.3

**Fig. 1:**
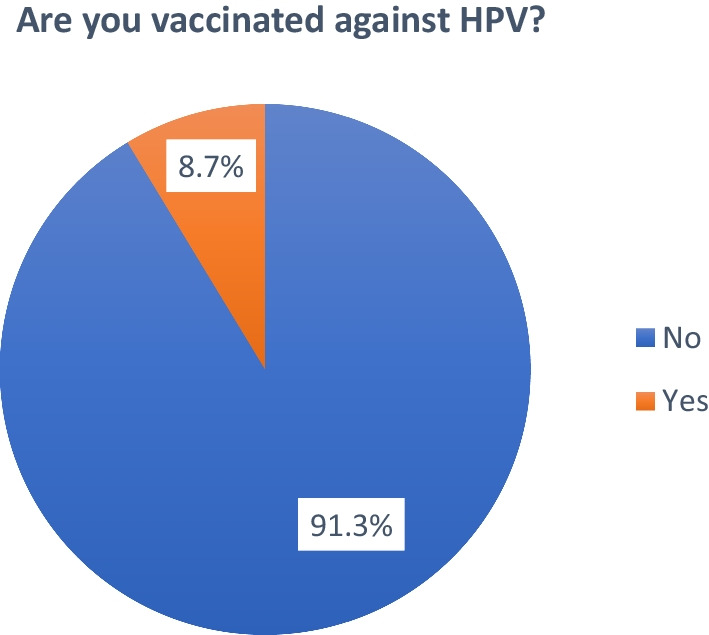
Prevalence of vaccination in the study population. Children's data were further collected. Children were distributed between 161 (41.1%) males and 231 (58.9%). The average age of children was 13.3 ± 2.7 years with a minimum of nine years and a maximum of 17 years. The average number of siblings was 1.7 ± 1.01. As per the parents, only 4.1% confirmed that the child insurance covers the HPV vaccine, 88.8% confirmed that their child took all of their early childhood vaccinations (mandatory vaccinations), and 44.4% stated that their child had an adverse effects post-vaccination (Table 4)

Furthermore, out of 392 participants, 34 (8.7%) were vaccinated against HPV and 358 (91.3%) were not vaccinated against HPV as shown in Fig. 1.

**Table 4:** Children related characteristics (N = 392)

		Frequency	Percent
Sex of your child to whom the questionnaire is about	Male	161	41.1
	Female	231	58.9
Age	Mean (SD)	13.3 (2.7)	
	Median	13	
	Min–Max	9–17	
Total number of siblings of the child?	Mean (SD)	1.7 (1.0)	
	Median	2	
	Min–Max	0–5	
Your child's school grade?	Grade 3	40	10.2
	Grade 4	43	11.0
	Grade 5	34	8.7
	Grade 6	29	7.4
	Grade 7	39	9.9
	Grade 8	36	9.2
	Grade 9	34	8.7
	Grade 10	19	4.8
	Grade 11	19	4.8
	Grade 12	99	25.3
Does your child’s insurance cover the HPV vaccine	No	172	43.9
	Yes	16	4.1
	Don't know	204	52
Did your children take all of their early childhood vaccinations (mandatory vaccinations)?	No	18	4.6
	Yes	348	88.8
	Not sure	26	6.6
Did your child have an adverse effects post-vaccination	No	218	55.6
	Yes, normal adverse effects (vaccine injection pain, low-grade fever, fatigue)	173	44.1
	Yes, severe adverse effects (inability to walk, trouble of breathing, high-grade fever, total body rash, severe illness)	1	0.3

On the other hand, out of 392 children, only 12.8% took the HPV vaccine previously of whom 44% took two doses, 24% took three doses and 32% took only one dose. The average age of child vaccination was 12.8 ± 1.9 years. The vaccine was recommended was the pediatrician (74%), the physician (14%), and by a friend (4%). Out of 392 parents, 66% read about the HPV vaccine before giving it to their child (Table 5).

**Table 5:** Children's vaccination against HPV (N = 392)

		Frequency	Percent
Did any of your children took the HPV vaccine previously?	No	289	73.7
	Yes	50	12.8
	Not sure	53	13.5
How many doses did he/she take	One	16	32.0
	Two	22	44.0
	Three	12	24.0
At what age did your child have his/her first dose	Mean (SD)	12.8 (1.9)	
	Median	12.5	
	Min–Max	10–17	
Who recommended that your child receive the vaccine	Your child pediatrician	37	74.0
	Your doctor	7	14.0
	You had heard about it from friends or family and asked the doctor for it	2	4.0
	Your own decision	4	8.0
You read about the HPV vaccine before giving it to your child	No	17	34.0
	Yes	33	66.0

**Fig. 2:**
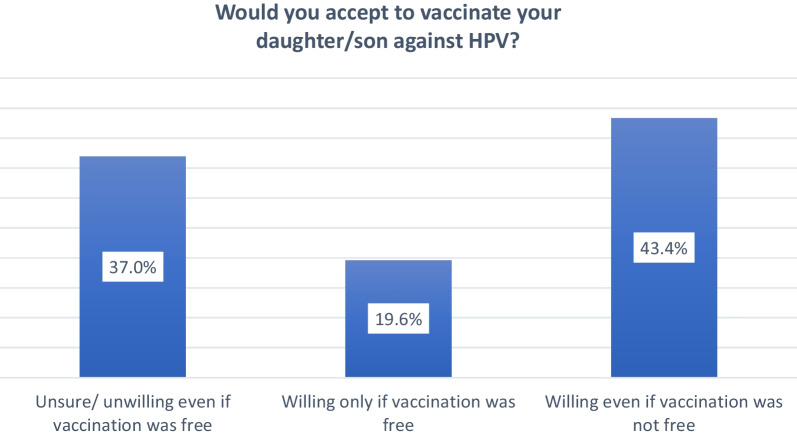
Intention to vaccination against HPV (N = 392)

The respondents were further interviewed regarding their willingness to vaccinate their children and their perception of HPV vaccination. Among the 392 parents, 63% will give their child the vaccine against HPV (will of vaccination is free or even if it was not free), whereas 37% are not willing or unsure to vaccinate their child against HPV (Fig. 2).

Our results demonstrated that out of 392 parents, 15.7% believe their daughter will be fully protected against cervical cancer after HPV vaccination. The top reasons for not vaccinating their child were (1) the vaccine was not offered (29.6%), (2) never heard about the vaccine (21.9%), and (3) the child is too young (20.9%). In addition, 5.9% are concerned that their daughter would have more sexual partners or practice more unsafe sex (i.e., not use a condom) if she will receive the vaccine, and 15.6% have concern about sexual partners. These findings are summarized in Table 6.

**Table 6:** Perceptions about vaccination against HPV (N = 392)

		Frequency	Percent
Do you believe your daughter will be fully protected against cervical cancer after HPV vaccination	No	103	30.5
	Yes	53	15.7
	Don't know	182	53.8
My child has not received the vaccine because	It was not offered to us	116	29.6
	I don’t think she needs it	24	6.1
	It is too expensive	34	8.7
	I don’t want to give my child an extra shot	24	6.1
	Never heard of it before	86	21.9
	We are against vaccinations	3	0.8
	This vaccine has many side effects	19	4.8
	My child is still too young	82	20.9
	It is not necessary for boys	36	9.2
Are you concerned that your daughter would have more sexual partners or practice more unsafe sex (i.e., not use a condom)? If she was vaccinated against HPV?	No	178	45.4
	Yes	23	5.9
	Don't know	121	30.9
	Do not want to answer	70	17.9
Would the concern above make you abstain from vaccinating your daughter against HPV?	No	178	45.4
	Yes	61	15.6
	Don't know	105	26.8
	Do not want to answer	48	12.2

Knowledge about HPV was assessed using 16 items and a detailed descriptive analysis about the correct answers is represented in Table 7. Results showed that parents had a lack of knowledge about the majority of HPV statements.

**Table 7:** Knowledge about HPV (N = 392)

		Frequency	Percent	% of correct answers
1. HPV is very rare	TRUE	64	16.3	36.0
	FALSE *	141	36.0	
	Don't Know	187	47.7	
2. HPV always has visible signs or symptoms	TRUE	46	11.7	32.1
	FALSE *	126	32.1	
	Don't Know	220	56.1	
3. HPV can cause cervical cancer	TRUE *	192	49.0	49.0
	FALSE	26	6.6	
	Don't Know	174	44.4	
4. HPV can be transmitted through genital skin-to-skin contact	TRUE *	135	34.4	34.4
	FALSE	73	18.6	
	Don't Know	184	46.9	
5. There are many types of HPV	TRUE *	156	39.8	39.8
	FALSE	6	1.5	
	Don't Know	230	58.7	
6. HPV can cause HIV/AIDS	TRUE	56	14.3	28.6
	FALSE *	112	28.6	
	Don't Know	224	57.1	
7. HPV can be passed on during sexual intercourse	TRUE *	210	53.6	53.6
	FALSE	27	6.9	
	Don't Know	155	39.5	
8. HPV can cause genital warts	TRUE *	139	35.5	35.5
	FALSE	14	3.6	
	Don't Know	239	61.0	
9. Men cannot get HPV	TRUE	49	12.5	37.5
	FALSE *	147	37.5	
	Don't Know	196	50.0	
10. Using condoms reduces the chances of HPV transmission	TRUE *	200	51.0	51.0
	FALSE	20	5.1	
	Don't Know	172	43.9	
11. HPV can be cured with antibiotics	TRUE	28	7.1	31.4
	FALSE *	123	31.4	
	Don't Know	241	61.5	
12. Having many sexual partners increases the risk of getting HPV	TRUE *	220	56.1	56.1
	FALSE	17	4.3	
	Don't Know	155	39.5	
13. HPV usually doesn't need any treatment	TRUE *	32	8.2	8.2
	FALSE	149	38.0	
	Don't Know	211	53.8	
14. Most sexually active people will get HPV at some point in their lives	TRUE *	94	24.0	24.0
	FALSE	70	17.9	
	Don't Know	228	58.2	
15. A person could have HPV for many years without knowing it	TRUE *	167	42.6	42.6
	FALSE	20	5.1	
	Don't Know	205	52.3	
16. Having sex at an early age increases the risk of getting HPV	TRUE *	145	37.0	37.0
	FALSE	37	9.4	
	Don't Know	210	53.6	

*Correct answer

Furthermore, knowledge about HPV was evaluated via a score which was ranged between 0 and 16. A reliability test was enrolled and results showed that the score is strongly validated in the study population with a Cronbach alpha value of 0.933, KMO (Kaiser–Meyer–Olkin Measure of Sampling Adequacy) equal to 0.952, and a significate result of Bartlett's Test of Sphericity (p < 0.0001).

The average knowledge score was 5.9 ± 4.8 over 16, median score was 6 over 16 with a minimum of 0 over 16 and a maximum of 16 over 16 (Table 8). Therefore, findings showed low knowledge of parents about HPV.

**Table 8:** Evaluation of knowledge about HPV (N = 39

		Knowledge about HPV
N		392
Mean		5.9
Median		6.0
Std. Deviation		4.8
Minimum		0
Maximum		16
Percentiles	25	0.0
	50	6.0
	75	10.0

Knowledge about HPV vaccine was assessed using 7 items and a detailed descriptive analysis about the correct answers is represented in Table 9. Results showed that parents had a lack of knowledge about the majority of HPV vaccine statements.

**Table 9:** Knowledge about HPV vaccine (N = 392)

		Frequency	Percent	Percent
1. HPV vaccine requires only one dose	TRUE	27	6.9	43.9
	FALSE *	172	43.9	
	Don't Know	193	49.2	
2. The HPV vaccine offers protection against all sexually transmitted infections	TRUE	57	14.5	40.3
	FALSE *	158	40.3	
	Don't Know	177	45.2	
3. The HPV vaccine is most effective if given to people who've never had sex	TRUE *	149	38.0	38.0
	FALSE	47	12.0	
	Don't Know	196	50.0	
4. Someone who has had the HPV vaccine cannot develop cervical cancer]	TRUE	58	14.8	30.9
	FALSE *	121	30.9	
	Don't Know	213	54.3	
5. The HPV vaccine offers protection against most cervical cancers	TRUE *	164	41.8	41.8
	FALSE	42	10.7	
	Don't Know	186	47.4	
6. The HPV vaccine offers protection against genital warts	TRUE *	111	28.3	28.3
	FALSE	35	8.9	
	Don't Know	246	62.8	
7. Girls who have had the HPV vaccine do not need a Pap test when they are older	TRUE	21	5.4	38.3
	FALSE *	150	38.3	
	Don't Know	221	56.4	

*Correct answer

Knowledge about the HPV vaccine was evaluated via a score which was ranged between 0 and 7. Reliability test was enrolled and results showed that the score is strongly validated in the study population with a Cronbach alpha value of 0.851, KMO equal to 0.892, and a significant result of Bartlett's Test of Sphericity (p < 0.0001).

The average knowledge score was 2.6 ± 2.2 over 7, median score was 3 over 7 with a minimum of 0 over 7 and a maximum of 7 over 7 (Table 10). Therefore, findings showed low knowledge of parents about the HPV vaccine.

**Table 10:** Evaluation of knowledge about HPV vaccine (N = 392)

		Knowledge about vaccine
N		392
Mean		2.6
Median		3.0
Std. Deviation		2.2
Minimum		0
Maximum		7
Percentiles	25	0.0
	50	3.0
	75	4.0

Bivariate analysis was enrolled to test the association between the “Intention to vaccination against HPV” and all the secondary variables. The results of the bivariate analysis were shown in Table 11.

**Table 11:** Factors associated with the willingness to vaccinate the children

		Intention to vaccination against HPV		Total (N = 392)	P value
		Unsure/unwilling (N = 145)	Willing (N = 247)		
Mother's nationality	Lebanese	133	243	376	**0.002**
		91.7%	98.4%	95.9%	
	Others	12	4	16	
		8.3%	1.6%	4.1%	
Father's nationality	Lebanese	135	237	372	0.216
		93.1%	96.0%	94.9%	
	Others	10	10	20	
		6.9%	4.0%	5.1%	
Mother's age	Mean (SD)	42.1 (5.7)	42.7 (5.3)	42.5 (5.4)	0.287
	Min–Max	27–54	22–55	22–55	
Father's age	Mean (SD)	47.63 (5.8)	47.93 (5.8)	47.82 (5.8)	0.630
	Min–Max	32–60	25–60	25–60	
Mother's educational level	No formal Education	13	9	22	0.112
		9.0%	3.6%	5.6%	
	School-level Education	37	57	94	
		25.5%	23.1%	24.0%	
	University Diploma	68	123	191	
		46.9%	49.8%	48.7%	
	Higher Education (Masters, PhD)	27	58	85	
		18.6%	23.5%	21.7%	
Father's educational level	No formal Education	19	12	31	**0.032**
		13.1%	4.9%	7.9%	
	School-level Education	44	84	128	
		30.3%	34.0%	32.7%	
	University Diploma	57	110	167	
		39.3%	44.5%	42.6%	
	Higher Education (Masters, PhD)	25	41	66	
		17.2%	16.6%	16.8%	
Mother's profession	Medical	26	39	65	0.582
		17.9%	15.8%	16.6%	
	Non-medical	119	208	327	
		82.1%	84.2%	83.4%	
Father's profession	Medical	6	20	26	0.128
		4.1%	8.1%	6.6%	
	Non-medical	139	227	366	
		95.9%	91.9%	93.4%	
Mother's religion	Christian	125	212	337	0.917
		86.2%	85.8%	86.0%	
	Muslim	20	35	55	
		13.8%	14.2%	14.0%	
Father's religion	Christian	122	213	335	0.570
		84.1%	86.2%	85.5%	
	Muslim	23	34	57	
		15.9%	13.8%	14.5%	
Residence	Beirut	44	56	100	0.220
		30.3%	22.7%	25.5%	
	Mount Lebanon	87	151	238	
		60.0%	61.1%	60.7%	
	South Lebanon	4	9	13	
		2.8%	3.6%	3.3%	
	North Lebanon	2	3	5	
		1.4%	1.2%	1.3%	
	Beqaa	8	28	36	
		5.5%	11.3%	9.2%	
Marital status	Married	135	233	368	0.624
		93.1%	94.3%	93.9%	
	Widow	10	14	24	
		6.9%	5.7%	6.1%	
Family income per month	Less than 2.000.000	17	12	29	**0.046**
		11.7%	4.9%	7.4%	
	Between 2.000.000–4.000.000	31	47	78	
		21.4%	19.0%	19.9%	
	Between 4.000.000–8.000.000	39	65	104	
		26.9%	26.3%	26.5%	
	More than 8.000.000	58	123	181	
		40.0%	49.8%	46.2%	
Do you have any history of cancer	No	121	190	311	0.123
		83.4%	76.9%	79.3%	
	Yes	24	57	81	
		16.6%	23.1%	20.7%	
Do you have any history of "cervical" cancer in your family	No	133	228	361	0.836
		91.7%	92.3%	92.1%	
	Yes	12	19	31	
		8.3%	7.7%	7.9%	
Have you ever heard of the Human papillomavirus (HPV) vaccine	No	81	80	161	** < 0.001**
		55.9%	32.4%	41.1%	
	Yes	64	167	231	
		44.1%	67.6%	58.9%	
Are you vaccinated against HPV	No	140	218	358	**0.005**
		96.6%	88.3%	91.3%	
	Yes	5	29	34	
		3.4%	11.7%	8.7%	
Sex of your child to whom the questionnaire is about	Male	69	92	161	**0.045**
		47.6%	37.2%	41.1%	
	Female	76	155	231	
		52.4%	62.8%	58.9%	
Does your child's insurance cover the HPV vaccine	No	48	124	172	** < 0.001**
		33.1%	50.2%	43.9%	
	Yes	3	13	16	
		2.1%	5.3%	4.1%	
	Don't know	94	110	204	
		64.8%	44.5%	52.0%	
Did your children take all of their early childhood vaccinations (mandatory vaccinations)?	No	11	7	18	**0.001**
		7.6%	2.8%	4.6%	
	Yes	117	231	348	
		80.7%	93.5%	88.8%	
	Not sure	17	9	26	
		11.7%	3.6%	6.6%	
Did your child have adverse effects post-vaccination	No	87	131	218	0.321
		60.0%	53.0%	55.6%	
	Yes, normal adverse effects (vaccine injection pain, low-grade fever, fatigue)	58	115	173	
		40.0%	46.6%	44.1%	
	Yes, severe adverse effects (inability to walk, trouble breathing, high-grade fever, total body rash, severe illness)	0	1	1	
		0.0%	0.4%	0.3%	
Knowledge about HPV	Mean (SD)	4.7 (4.6)	6.7 (4.8)	5.9 (4.8)	** < 0.001**
	Min–Max	0–16	0–16	0–16	
Knowledge about Vaccine	Mean (SD)	1.9 (2.0)	3.0 (2.2)	2.6 (2.2)	** < 0.001**
	Min–Max	0–7	0–7	0–7	

Significant p values are shown in bold

Findings showed that there was a statistically significant correlation between the “Intention to vaccination against HPV” and mother’s nationality (p = 0.002), father's educational level (p = 0.032), family income per month (p = 0.046), received information about the Human Papilloma Virus (HPV) vaccine (p < 0.001), parents’ vaccination against HPV (p = 0.005), insurance coverage of HPV vaccine (p < 0.001), sex of the children (p = 0.045), childhood vaccinations of all mandatory vaccines (p = 0.001), knowledge about HPV (p < 0.001) and knowledge about HPV vaccine (p < 0.001).

Findings showed that intention to vaccination was more prevalent in Lebanese mothers (98.4%). In addition, results showed the will to vaccinate the child against HPV was increasing with the increasing of fathers’ educational level where the positive will to vaccination increased from 4.9% in fathers with no formal education to 44.5% in fathers with a university level. Findings showed the will to vaccinate the child against HPV was increasing with the increase of the monthly income where the positive will to vaccination increased from 4.9% in families with low monthly income to 49.8% in families with high monthly income.

Having information about the HPV vaccine was initial in the positive intention to vaccination. Intention to vaccination was more prevalent in parents who had ever heard about the HPV vaccine (67.6%) whereas 55.9% of parents who had never heard about the HPV vaccine had negative intention to vaccination against HPV.

Findings showed that intention to vaccination was more prevalent in parents who were previously vaccinated against HPV, in parents who provided their child with all the mandatory vaccines and in parents who confirmed that the insurance covers the HPV vaccine. Intention to vaccination against HPV was higher in parents who have female children (62.8%) compared to parents who have boy children (p = 0.045).

Knowledge about HPV was higher in parents who are willing to vaccinate their child (average knowledge score was 6.7 ± 4.8) compared to parents who are not willing to vaccinate their child (average knowledge score was 4.7 ± 4.6).

Knowledge about the HPV vaccine was higher in parents who are willing to vaccinate their child (average knowledge score was 3.0 ± 2.2) compared to parents who are not willing to vaccinate their child (average knowledge score was 1.9 ± 2).

Binary logistic analysis was conducted to assess the factors associated with the willingness to vaccinate the children. The model included the dependent variable “Intention to vaccination against HPV” and the independent variables which were associated with the dependent variable in the bivariate settings. After adjusting the model, the final findings with the adjusted Odds Ratio are represented in Table 12.

**Table 12:** Binary logistic analysis for the factors associated with the willingness to vaccinate the children

	B	S.E	p.value	Exp(B)	95% C.I.for EXP(B)	
					Lower	Upper
Have you ever heard of the Human papillomavirus (HPV) vaccine	0.606	0.241	0.012	1.832	1.141	2.941
Knowledge about Vaccine	0.188	0.057	0.001	1.207	1.079	1.351

Findings showed that two factors increase the will to vaccinate the child against HPV noting: having information about the HPV vaccine (p = 0.012) and knowledge (score) about the HPV vaccine (p = 0.001). The will to vaccinate the child against HPV increased 1.832 times (95% CI [1.141–2.941] when the parents had information about HPV and increased 1.207 times (95% CI [1.079–1.351] when the parents’ knowledge score increased one point.

## Discussion

Our study explored the overall intention of Lebanese mothers to vaccinate their children against HPV among 392 participants. The majority of mothers (95.9%) and fathers (94.9%) were Lebanese and the average ages of mothers and fathers were 42.5 ± 5.4 years and 47.8 ± 5.8 years respectively. The average age of children was 13.3 ± 2.7 years with a minimum of nine years and a maximum of 17 years.

### History of cervical cancer

Concerning cervical cancer, 20.7% of the participants had a history of cancer, about 4.3% had a history of cervical cancer, and 7.9% had a family history of cervical cancer. In another study conducted in Lebanon, 6.7% of the participants declared to have a family history of cervical cancer [19], which is roughly equivalent to what our study has proven. Another study's findings revealed that 12.8% of participants had a history of other cancers, and 1.3% of participants had a family history of cervical cancer [26]. The little discrepancy might be brought on by the different sample sizes. Therefore, given that they have been exposed to this malignancy or have a family history of it, research participants should be aware about cervical cancer and how to prevent it.

### HPV vaccination

It was essential to evaluate the percentage of knowledge about the HPV vaccine and the rate of vaccination among participants 231 participants (58.9%) had ever heard of the Human Papilloma Virus (HPV) vaccine from different sources of information. There are various general communication approaches accessible to everyone to provide knowledge about HPV [17], and the top four sources of information in our survey were Pediatricians (32.5%), physicians (22.9%), Media/Internet (19.9%), and family (10%). Parents appear to trust information provided by caregivers more than that shared on social media or any other means.

Out of 392 participants, only 34 (8.7%) participants were vaccinated against HPV. Moreover, it was critical to understand parents’ willingness to have their children vaccinated against HPV. Among the 392 parents surveyed, 43.4% would give their child the HPV vaccine even if it was not free, while 19.6% would only vaccinate them if it was free. However, 37% are unwilling or uncertain about immunizing their child against HPV. In a cross-sectional school-based survey, 14.4% of mothers of unvaccinated children choose not to vaccinate their daughters because of the expensive cost; and they would be more likely to do so if it were free [19].

### Knowledge about HPV and HPV vaccination

Furthermore, several studies have shown that one of the most significant factors affecting HPV vaccination rates is parents' knowledge and awareness regarding HPV infection and the corresponding vaccine [27]. Indeed, Abou El Ola et underlined in their study that one of the challenges to HPV vaccination is a lack of parental understanding of cervical cancer and the HPV vaccine [19].

Our study showed that parents lack knowledge about the majority of statements about HPV infection and the HPV vaccine. These results are consistent with other studies. In fact, in a pre-post structured-educational intervention study conducted by Sitaresmi et al., the parental awareness about HPV and HPV vaccine has been demonstrated to be insufficient (< 50%) [28]. Only 46.2% of parents have heard about HPV infection and about 44.1% of them have heard about HPV vaccination [28]. Thus, many future approaches and campaigns should be addressed to increase the level of awareness and knowledge regarding HPV infection and HPV vaccine among parents.

On the other hand, knowledge regarding HPV and the HPV vaccine was dramatically insufficient in a study conducted in Morocco. Their analysis indicates that only 4.7% of participants were aware of HPV infection and 14.3% were aware of the HPV vaccination [29].

### Factors affecting parent’s willingness to vaccinate their children

To achieve the aim of our study, a bivariate analysis was conducted to test the association between the “Intention to vaccination against HPV” and all the secondary variables.

Our results showed that there was a statistically significant correlation between the “Intention to vaccination against HPV” and the mother’s nationality. Indeed, the intention to vaccinate was more prevalent in Lebanese mothers than in others. This is at some point compatible with what has been demonstrated by Abou El Ola et al. Indeed, their study underlined a significant correlation between the awareness regarding the HPV vaccine and being a Lebanese mother [19]. From there, it seems necessary to educate not only Lebanese mothers but also mothers of other nationalities because they are part of society.

Concerning parent’s educational level, it has already been demonstrated that when parents are more educated, their intention to vaccinate their children is greater. This implies that parents' educational degree influences their willingness to vaccinate their children [30]. However, our study showed that the intention of vaccination is significantly associated with the father’s educational level, and not that of the mother. Indeed, the positive will toward vaccination increased from 4.9% in fathers with no formal education to 44.5% in fathers at the university level. These results are in line with the findings of Destaw et al. [30] but contradict the findings of Mouallif et al. who showed that children's vaccination acceptance was significantly associated with the mother's education level, but no significant association was found with the fathers' educational level [29].

On the other hand, a positive significant correlation was found between monthly income and vaccination acceptance. Indeed, the participants with higher monthly income have a higher intention to vaccinate their children. Consistently with our results, a study done in Morocco has demonstrated that parents with a medium to high monthly income were more willing to accept vaccination than parents with low income [29].

Additionally, Findings showed that there was a statistically significant correlation between the “Intentions to vaccinate against HPV” and receiving information about the Human papillomavirus (HPV) vaccine (p < 0.001). Indeed, being aware and having information about the HPV vaccine was initial in the positive intention to vaccination. Also, parents who are already vaccinated against HPV, thus having a high knowledge and awareness, have a significantly higher intention to vaccinate their children. Studies have underlined that receiving positive information concerning the HPV vaccine is essential to improve the parent’s acceptance of vaccination [23], indeed parents who had heard about the HPV vaccine were more willing to consent to vaccination than parents who were unfamiliar with it [29].

One of the potential barriers to HPV vaccination is the lack of Knowledge. In this analysis, we were able to prove that parents' willingness to vaccinate their child was significantly higher in parents with higher knowledge about HPV (p < 0.001) and higher Knowledge about HPV vaccine (p < 0.001). Similarly, studies have demonstrated that one of the most important factors that negatively affect the parent’s acceptance of vaccination is their lack of knowledge regarding HPV, HPV vaccine, and cervical cancer [19, 20, 23]. Therefore, increasing parents' knowledge and ensuring that they receive adequate information regarding HPV, the HPV vaccine, and cervical cancer will inevitably lead to an increase in the vaccination rate.

Moreover, parents with insurance coverage of the HPV vaccine and those who already gave their children all childhood mandatory vaccines were more likely to accept the vaccination (p < 0.001). Generally, a qualitative systematic review conducted by Ferrer et al. demonstrated that the vaccine cost is one of the barriers preventing people from vaccination [31]. Moreover, another systematic review showed that the high cost of the vaccine is associated with a lower intention of parents to have their children vaccinated [32]. This explains the logic of our results, since the cost of the vaccine is a barrier, thus once the cost of the vaccine is covered by the insurance, parents are significantly more accepting of vaccination. Consequently, it seems necessary to provide the vaccine for free or at a low cost.

Parents who had already given their children all the compulsory childhood vaccinations were more likely to accept the vaccination, as they were no longer afraid of several factors. Indeed, Mihretie et al. demonstrated that some parents do not accept the HPV vaccine due to the fear of side effects and the fear of needle infection [20].

Yet, no significant correlation was found between “Parents' willingness to vaccinate their child” and the father’s nationality, parents' age, mother's educational level, parent's profession, parent's religion, residence, marital status, children sex, history of cervical cancer and post-vaccination adverse effects. However, literature showed that younger mothers are more likely to accept to vaccination of their children than older ones [20], and that religion continued to have a significant role in HPV vaccination acceptance, with girls from Muslim or Hindu/Sikh families being less likely to receive vaccination [33].

Similar to these results, Han and Son showed no significant relationship between mother’s decision to vaccinate their children and age, marital status, educational level, religion, and family history of cervical cancer [23].

A binary logistic analysis was then performed to evaluate the variables influencing the parent's desire to vaccinate their child. Our findings revealed that receiving information about the HPV vaccine and knowledge (score) about the HPV vaccine increase parent's willingness to vaccinate their kid against HPV. When parents have information about HPV, their willingness to vaccinate their child rises by a factor of 1.832 times, and by 1.207 times when their knowledge level increases by one point.

In line with our findings, many researchers have proven that parents who have already heard about the HPV vaccine and those with good knowledge about the HPV vaccine have an increased intention to vaccinate their children. Sinshaw et al. conducted a community-based cross-sectional study with a total sample of 601 study participants to evaluate the knowledge and attitude toward the HPV vaccine among mothers who have eligible girls. When compared to moms who had inadequate knowledge about the HPV vaccination, mothers with strong knowledge were demonstrated to be three times more likely to accept vaccinating their daughters [34]. Similarly, Destaw et al. underlined a positive correlation between having good knowledge about the HPV vaccine and parents’ intention to vaccinate their children [30]. Moreover, Mihretie et al. stated that parents who know about HPV vaccine and cervical cancer have 3.30 times higher acceptability of vaccinating their children than those with insufficient knowledge [20].

In conclusion, many factors are associated with parents’ acceptance to vaccinate their children. Most importantly, knowledge about HPV vaccination and the availability of the vaccine at a lower cost seems to play an important role in increasing the vaccination rate among adolescents, not only in Lebanon but Worldwide. Our findings highlight the critical need for increasing HPV awareness and vaccination among Lebanese parents. The prevalence of vaccination misconceptions and the lack of understanding about HPV, highlight the significance of more comprehensive sexual education in schools, as well as the spread of accurate information about HPV and its vaccine. Given that non-Lebanese mothers have poor knowledge of HPV and the number of Syrians in Lebanon is relatively large, efforts should be made to encourage HPV vaccination among this group. Addressing these concerns can enhance public health and aid in the prevention of HPV and its associated disorders.

### Strengths and limitations

The cross-sectional study methodology and the use of a relatively large sample size might be considered as the study's strengths. Furthermore, this is the first study in Lebanon addressing the willingness of Lebanese parents to vaccinate their children, as they are known to be the main decision-makers in adolescent immunization. It also sheds light on the importance of implementing systematic educational programs all over Lebanon to improve parents' knowledge about HPV and HPV vaccination. Our research enables the collection of enormous amounts of data with a single survey. This form of survey is cost-effective, time-effective, and high-yielding.

Nevertheless, this study has been conducted in the presence of different limitations. One of the main limitations of this study was the limited access to the study population. Indeed, many schools and educational centers refused to participate in this study and a large proportion of asked parents were not cooperative and refused to participate which has affected the study process. It took us a longer time to reach the required sample. Study limitations would be also due to social desirability bias.

Future studies should be addressed with a higher number of participants in cooperation with third parties and educational centers.

## Conclusion

The findings of this study showed that parents had a lack of knowledge about most HPV-related statements. A statistically significant positive correlation was demonstrated between the “Intention to vaccination against HPV” and having information about the HPV vaccine and the knowledge about the HPV vaccine.

Parental awareness, knowledge, and attitudes regarding HPV as well as their willingness to vaccinate their children may be enhanced by a systematic educational intervention and multimodal approaches that address the barriers. The study made it possible to identify the factors that lead to a limited vaccination rate in Lebanon, which will help address these factors in subsequent campaigns, suggesting the possibility of using such cross-sectional surveys as intervention tools.

In conclusion, while a few researchers have evaluated knowledge and perceptions concerning HPV vaccination among Lebanese parents, the literature appears to lack an in-depth assessment of obstacles and facilitators further than typical survey measures, for HPV vaccine availability, rate among adolescents, and parents’ willingness to vaccinate their children. Therefore, further studies on larger samples are recommended.

## Data Availability

The datasets generated and analyzed during the current study are not publicly available but are available from the corresponding author upon reasonable request.
